# Longer theoretical length of cut in whole-plant sorghum silage increased feed intake and starch digestibility in growing beef steers

**DOI:** 10.1093/tas/txag077

**Published:** 2026-06-03

**Authors:** Federico Fernandez, Federico Tarnonsky, Adrian Rodriguez-Guiñazu, Ruben Arias, Jorge Delgado-Caffé, Leandro O Abdelhadi, Nicolas DiLorenzo

**Affiliations:** Facultad de Ciencias Agrarias y Forestales, Universidad Nacional de La Plata, La Plata, 1900, Argentina; North Florida Research and Education Center, Institute of Food and Agricultural Sciences, University of Florida, Marianna, FL, 32446, United States; Facultad de Ciencias Agrarias y Forestales, Universidad Nacional de La Plata, La Plata, 1900, Argentina; Facultad de Ciencias Agrarias y Forestales, Universidad Nacional de La Plata, La Plata, 1900, Argentina; Facultad de Ciencias Agrarias y Forestales, Universidad Nacional de La Plata, La Plata, 1900, Argentina; Facultad de Ciencias Veterinarias, Universidad Nacional de La Plata, La Plata, 1900, Argentina; Facultad de Ciencias Agrarias y Forestales, Universidad Nacional de La Plata, La Plata, 1900, Argentina

**Keywords:** beef cattle, chop length, sorghum silage, starch digestibility, theoretical length of cut

## Abstract

This study investigated the growth performance of beef steers fed diets primarily composed of sorghum silage differing in theoretical length of cut (TLOC). Seventy-two Angus steers (200 ± 40 kg of initial body weight) were housed in 12 pens (6 steers/pen) and utilized in a randomized complete block design for an 84-d feeding trial. The experiment compared two dietary treatments (6 pens/treatment): one containing 89% sorghum silage in dry matter (DM) basis chopped at a 15 mm TLOC and another with 89% sorghum silage (DM basis) chopped at a 7.5 mm TLOC. Both diets were supplemented with 10% cracked soybeans and 1% mineral-vitamin premix. The results indicated that steers consuming the diet with the larger particle size (15 mm TLOC) exhibited greater dry matter intake (*P* = 0.05) compared to those fed the shorter cut (7.42 vs. 7.26 kg/d). Additionally, growing steers fed a high forage diet with a 15-mm TLOC silage showed greater starch digestibility (*P* = 0.03) compared to those fed the 7.5-mm TLOC diet (92.2% vs. 88.5%, respectively). Despite these differences in intake and digestibility, average daily gain and feed efficiency did not differ between treatments. These findings indicate that producers using conventional processors may optimize energy availability and feed intake by choosing a moderate chop length (15 mm) rather than cutting the forage too finely.

## Graphical abstract

**Graphical Abstract:**
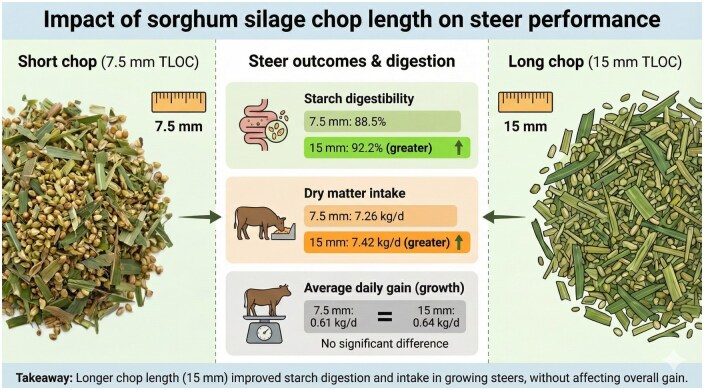

## Teaser

This study challenges the assumption that finer chopping always improves digestion, demonstrating that a longer theoretical length of cut (15 mm) in sorghum silage increases feed intake and starch digestibility in growing beef steers.

## Introduction

Sorghum (*Sorghum bicolor [L.]* Moench) is widely adopted as a forage crop in ruminant systems due to its agronomic resilience, particularly its drought and heat tolerance, which makes this crop a sustainable alternative to corn silage in water-limited environments and different types of soil (Adewakun et al. 1989). Sorghum silage has been proven as a viable alternative for various production systems due to adequate average daily gains of 0.5–1.0 kg in backgrounding cattle when properly supplemented with protein (Tarnonsky et al. 2023), and due to decreased production costs, enhanced water efficiency, and comparable yields to those with corn (Oliver et al. 2004). However, sorghum silage often exhibits lower energy density compared to corn, primarily due to its lower grain-to-stover ratio and particularly poor starch digestibility, as the small, dense kernels with hard, waxy pericarps resist both mechanical processing and ruminal degradation (Ferreira and Mertens 2005; Dias Junior et al. 2016; McCary 2019; McCary et al. 2020).

Mechanical processing at harvest is critical to improving kernel breakage and therefore starch digestibility (Ferraretto et al. 2018). Research shows that kernel processing increases starch digestibility while reducing fecal starch and enhancing NDF digestibility in whole-plant sorghum silage (Duhatschek et al. 2025; Podversich et al. 2025). Moreover, the theoretical length of cut (TLOC) plays a pivotal role, affecting packing density, fermentation quality, and forage structure when chopping corn silage within 9 and 19 mm (Ferraretto and Shaver 2012;  Ferraretto et al. 2018). Shorter TLOC aid in achieving finer packing, more rapid anaerobic fermentation, and improved aerobic stability, though this comes at the expense of physically effective fiber necessary for rumen health (Allen et al. 2000). In addition, increasing TLOC (ie chopping more coarsely) has been shown to reduce leachate production in silage, which can be a key consideration in humid forage systems (Messer and Hawkins 1977; Savoie et al. 2002). Despite the known benefits of kernel processing and TLOC adjustment, mainly in corn silage, there remains a research gap regarding their combined effects on animal performance and nutrient utilization in beef cattle fed sorghum silage. The hypothesis of this experiment was that chopping whole-plant sorghum silage to a finer particle with the use of a kernel processor at harvest would enhance intake and performance of beef steers driven by an enhanced grain fragmentation that would enhance intake and digestion of starch. Therefore, this study aimed to evaluate the impacts of two silages with differing TLOC (7.5 mm vs. 15 mm), both processed using a conventional kernel processor on feed intake, growth performance, and apparent total tract nutrient digestibility in backgrounding beef steers fed whole-plant sorghum silage as the main dietary ingredient.

## Materials and methods

All procedures were conducted according to the Guide for the Care and Use of Agricultural Animals in Agricultural Research and Teaching (FASS 2020) and with the approval of the Institutional Ethics and Security Committee (Protocol No. 107-2-20P, Facultad de Ciencias Veterinarias, Universidad Nacional de La Plata, Buenos Aires, Argentina). This experimental farm provided the plant material, the animals, and the facilities for the experiment.

### Plant material

The whole-plant silage was obtained from 9 ha of grain sorghum hybrid ADV1250IG (Advanta Seeds, Irving, TX), planted on December 23, 2019, at a rate of 200,000 seeds per hectare. The harvest was conducted on May 19, 2020, at a hard-dough stage, after an early freeze event. Low temperatures during the final growth period, combined with a Sugarcane Aphid infestation, resulted in a forage with reduced starch concentration at harvest. The forage was harvested by a Claas Jaguar 870 (Harsewinkel, Germany) harvester, equipped with a conventional sorghum kernel processor of 196-mm diameter rolls, with 110 teeth in both rolls, operating at a speed differential of 50% and to a roll gap of 1 mm. The 9-ha field was harvested on the same day, with TLOC randomly alternated across chopper passes. Specifically, TLOC settings were alternated systematically throughout the harvest. This approach ensured that both TLOC treatments were equally represented across the varying soil and growing conditions of the entire field. Both harvested plant materials were treated with a homofermentative silage inoculant (Ecosyl, Volac International Limited, Byron, IL) to provide a minimum of 100,000 colony forming units of *Lactobacillus plantarum* bacteria per gram of wet forage, and were stored in separate side-by-side 2.74-m-diameter silo bags and allowed to ferment for 30 d prior to the feeding trial. The silage material was bagged in two different bags, one per theoretical length of cut, and stored for 77 days until the beginning of the trial.

### Animals and diets

Seventy-two newly weaned steers (7 ± 2 months of age, 200 ± 40 kg of body weight, BW) were used in a randomized complete block design. Animals were blocked by body weight into 6 blocks. During the experimental period, 72 steers were housed in 12 pens of 100 m^2^ of 6 steers each, with free access to feed and water. Treatments consisted of a common sorghum silage-based diet [DM basis; 89% sorghum silage, 10% cracked soybeans, and 1% mineral-vitamin premix (vitamin A: 1,000,000 IU/kg, vitamin D3: 200,000 IU/kg, vitamin E: 6500 IU/kg, vitamin B1: 650 ppm, manganese: 12,000 ppm, zinc: 12,000 ppm, copper: 6000, cobalt: 40 ppm, selenium: 60 ppm, iodine: 200 ppm; Vetifarma, Buenos Aires, Argentina) formulated to differ specifically in the theoretical length of cut (7.5 vs. 15 mm)]. Diets were formulated to provide protein, vitamins, and minerals to meet steer requirements (NASEM 2016) with a target gain of 0.5 kg/d. Ingredient composition of the diets is shown in Table 1. The experimental period consisted of 14 days of adaptation followed by 84 days of intake and body weight change measurements every 28 days.

**Table 1:** Analyzed^a^ chemical composition of the ingredients^b^ (DM basis) of diets fed to Angus steers and fermentation parameters of the silages that composed 89% of the DM of the dietary treatments.

	Ingredient		
Item^c^	Cracked soybeans	Sorghum silage at 7.5 mm	Sorghum silage at 15 mm
**DM, %**	93.8	30.97	31.22
**Moisture, %**	6.2	69.03	68.78
**CP, % DM**	41.5	10.38	10.17
**Soluble CP, % DM**	–	51.65	52.57
**Ether extract, % DM**	19.33	2.16	2.04
**Ash, % DM**	6.28	7.78	8.88
**Starch, % DM**	–	14.8	13.14
**WSC, % DM**	–	0.28	0.37
**ADF, % DM**	7.83	30.88	31.97
**aNDF, % DM**	15.8	46.24	46.62
**Lignin, % DM**	–	4.84	4.89
**NDFD 30, % FDN**	–	46.17	44.41
**TTNDFD, % FDN**	–	40.11	38.81
***Fermentation Products***			
**pH**	–	4.15	4.13
**Lactic acid, % DM**	–	4.63	3.82
**Acetic acid, % DM**	–	0.88	0.71
**Butyric acid, % DM**	–	0.25	0.23
**N-NH_4_, %CP**	–	5.37	4.07
**uNDF 30**	–	24.9	25.9
**uNDF 240**	–	17.0	17.7

a Rock River Laboratory, Rosario, Argentina.

b Ingredients were sampled every two weeks for dietary DM corrections, and composited for analysis at the end of the experimental period.

c DM, dry matter; CP, crude protein; ADF, acid detergent fiber; aNDF, neutral detergent fiber corrected with alpha-amylase; WSC, water soluble carbohydrates; NDFD 30, neutral detergent fiber digestibility at 30 hours; TTNDFD, total tract neutral detergent fiber digestibility; N-NH_4_, ammonia nitrogen; uNDF 30, undegraded neutral detergent fiber at 30 hours; uNDF 240, undegraded neutral detergent fiber at 240 hours.

### Growth performance

Initial and final BW were collected after 16 hours of feed withdrawal. Interim full body weight measurements were taken every 28 days. Growth performance was assessed by calculating average daily gain (ADG), dry matter intake (DMI), and gain-to-feed ratio (G:F). Changes in BW were calculated by subtracting the initial BW measurement from the final BW. Pen-level DMI was calculated daily by subtracting orts from feed delivered. Feed delivery and refusals were corrected to a DM basis. The number of animals housed per pen was multiplied by number of days in the period to determine animal days, which were divided into the corrected total DM delivered to the pen to obtain average DMI per steer.

Steers were fed once daily with a horizontal mixing wagon (Martinez & Staneck MS-10, Buenos Aires, Argentina). Feed offered was weighed daily and recorded. Experimental diets were delivered at 0800 with a tractor-pulled mixer equipped with ± 10 kg precision scale. Chemical composition of the diets is shown in Table 2. Ingredients were sampled weekly to correct for DM differences and their proportions in the diet were adjusted on a DM basis, accordingly. Diet samples were collected from the bunks every 7 d, dried at 55°C for 72 h and composited by pen on an equal weight at the end of the experimental period. Composite diet samples were analyzed for nutritional composition by a commercial laboratory (Rock River Laboratory, Santa Fe, Argentina) through wet chemistry procedures for concentrations of crude protein (CP), neutral detergent fiber (NDF), acid detergent fiber (ADF), and starch. Fermentation products were determined by HPLC, while NDFD 30 and TTNDFD were estimated using the Daisy II incubator system (Ankom Technology Corp., Macedon, NY). The Penn State Particle Separator (PSPS) with sieves of 19.0, 8.0, 1.8 mm, and a pan was used (Kononoff et al. 2003; Heinrichs 2013) to characterize particle size distribution of the dietary treatments (Table 2) every two weeks during the 84-d performance trial. The geometric mean diameter (GMD) and the geometric standard deviation were calculated according to the (American Society of Agricultural and Biological Engineers Standards ASABE 2008) S319.4 standard based on the as-is weight of the material retained on each sieve of the PSPS. Calculations were performed using the log-normal distribution method, where the GMD was derived from the weighted sum of the logarithms of the sieve aperture midpoints to accurately represent the center of mass of the feed particles. The log-transformed midpoint diameters were weighted by multiplying them by their respective retained mass fractions. Then, the sum of these weighted values was divided by the total sample mass, and the resulting exponentiated value yielded the overall GMD of the particle distribution.

**Table 2:** Analyzed^a^ nutrient composition on a DM basis of dietary treatments^b^ fed to Angus steers.

Item^c^	Dietary treatment with sorghum silage at 7.5 mm	Dietary treatment with sorghum silage at 15 mm
**DM, % as is**	33.7	33.9
**CP, % DM**	10.9	11.0
**Ash, % DM**	7.47	8.4
**Starch, % DM**	11.75	12.65
**ADF, % DM**	27.95	27.85
**aNDF, % DM**	53.5	53.2
**Lignin, % DM**	4.45	4.50
***Particle size, % of DM retained***		
**> 19.0**	0.8%	6.0%
**19.0–8.0**	41.9%	51.1%
**8.0–1.8**	54.0%	40.8%
**<1.8**	3.3%	2.1%
**Geometric mean, mm**	6.96 ± 2.19	10.74 ± 2.40
**Estimated peNDF, %DM**	22.8	30.4

a Laboratorio de evaluación de calidad de forrajes y alimentos, INTA Bordenave, Argentina.

b Treatments were sampled every two weeks, and composited for analysis at the end of the experimental period.

c DM, dry matter; CP, crude protein; ADF, acid detergent fiber; aNDF, neutral detergent fiber corrected with alpha-amylase. Particle size was measured using the Penn State Particle Size Separator (Nasco, Fort Atkinson, WI) as described by Kononoff et al. (2003); estimated peNDF was estimated by multiplying percentage of particles greater than 8 mm by the dietary NDF.

### Apparent total tract digestibility of nutrients

Following the 84-d growth performance phase (preceded by a 14-d adaptation period), one steer per pen was randomly selected and individually housed in their respective same pens (100 m^2^ per pen) for 5 days. Due to limited amount of fecal samples from two steers during the 5-d collection period, each treatment ended up with 5 replicates. Feed was offered once daily during the collection period, and orts were collected the following day. Offered feed and orts were weighed daily using an independent scale. Feed samples were collected from days 0 to 4, while orts and fecal samples were collected from days 1 to 5 to determine DM and nutrient composition. Fecal samples were taken twice daily at 0800 and 1600 h for four consecutive days from rectal grab. Apparent total tract digestibility of dry matter, organic matter (OM), CP, starch, NDF, and ADF was measured in each pen using indigestible NDF (iNDF) as an internal marker. After collection, feed and fecal samples were immediately placed on ice until transport to the laboratory. Feed and fecal samples were then frozen at −20°C until further analysis. At the end of the experiment, feed and fecal samples were thawed and dried at 55°C for 72 h in a forced-air oven and then ground in a Willey mill (Arthur H. Thomas Co., Philadelphia, PA) to pass a 2-mm screen. Feed, orts, and fecal samples were composited on an equal weight basis, per animal, for determination of nutrient and internal marker concentration at the INTA Bordenave laboratory.

For feed and fecal analytical dry matter determination, 0.5 g of each sample were weighed in duplicate, dried in a forced-air oven at 100°C for 24 h, and ashed at 550°C for 6 h. For determination of the fibrous component, samples were weighed in duplicate into F57 bags (Ankom Technology Corp., Macedon, NY) and analyzed for neutral detergent fiber (aNDF) and acid detergent fiber (ADF) using an Ankom 200 Fiber Analyzer (Ankom Technology Corp). The aNDF analysis included the use of heat-stable alpha-amylase and sodium sulfite, following the procedures described by Goering and Van Soest (1970). Total nitrogen (N) concentration was determined according to the Kjeldahl method (AOAC 1990). Samples (100 mg) were digested with sulfuric acid and a copper/potassium sulfate catalyst mixture for 2.5 h, followed by distillation and titration with hydrochloric acid to determine N content. Total starch was determined using the enzymatic method (AACC Method 76-12; Megazyme International Ireland Ltd., Wicklow, Ireland). Samples were ground to 0.5 mm and washed with 80% ethanol to remove soluble sugars. The residue was treated with thermostable alpha-amylase in MOPS buffer (pH 7.0) at 100°C, followed by amyloglucosidase in acetate buffer (pH 4.5) at 50°C. Glucose concentration was measured colorimetrically at 510 nm using the glucose oxidase-peroxidase reaction. For indigestible NDF (iNDF) determination, 0.5 g of sample were weighed in duplicate into F57 bags (Ankom Technology Corp), incubated in the rumen of a cannulated steer consuming a 50:50 forage-to-concentrate diet for 288 h, rinsed with tap water until runoff was clear, dried at 60°C overnight, and incubated in an Ankom 200 Fiber Analyzer (Ankom Technology Corp) as described by Cole et al. (2011) with the modifications proposed by Krizsan and Huhtanen (2013). Apparent total tract digestibility of DM, NDF, ADF, starch and CP were calculated using the following formula:


$$100-100\times [\left(\frac{\mathrm{iNDF}\;\mathrm{concentration}\;in\;\mathrm{feed}}{\mathrm{iNDF}\;\mathrm{concentration}\;in\;\mathrm{feces}})\times (\frac{\mathrm{nutrient}\;\mathrm{concentration}\;in\;\mathrm{feces}}{\mathrm{nutrient}\;\mathrm{concentration}\;in\;\mathrm{feed}}\right)]$$

The percentage difference in NDF concentration between diet and refusals was calculated using the following formula:


$$100\times (\frac{\mathrm{NDF}\;\mathrm{concentration}\;\mathrm{in}\;\mathrm{refusals}-\mathrm{NDF}\;\mathrm{concentration}\;\mathrm{in}\;\mathrm{diet}}{\mathrm{NDF}\;c\mathrm{oncentration}\;in\;\mathrm{diet}})$$

### Statistical analysis

Data were analyzed using the MIXED Procedure of SAS (9.4 SAS Institute Inc., Cary, NC) as a randomized complete block design. Pen was the experimental unit (*n* = 6 per treatment) and the model included the fixed effects of treatment and the random effects of block and pen within treatment. Initial BW was tested as a covariate, remaining in the model when significant. For digestibility of nutrients, the MIXED procedure of SAS was used with animal as the experimental unit (*n* = 5 per treatment), treatment as a fixed effect, and block as a random effect. Starch intake was included as a covariate given the trend toward different starch intakes between treatments when starch digestibility was analyzed. The residuals and influencing diagnostic outputs from the MIXED procedure were checked for the assumptions of normality of the residuals and homogeneity of variance. Significance was declared at *P* ≤ 0.05, and tendencies were considered when 0.10 ≥ *P* > 0.05.

## Results

### Growth performance

Performance results are shown in Table 3. Initial BW did not differ between treatments (*P* = 0.43). Similarly, final body weight (*P* = 0.46) and average daily gain (*P* = 0.48) were not affected by TLOC. However, dry matter intake (DMI) differed between treatments, both when expressed in kg.day^−1^ (*P* = 0.05) and as a percentage of body weight (*P* = 0.05). Steers fed silage chopped at 15 mm of TLOC consumed 2.2% more DM than those fed silage chopped at 7.5 mm (7.42 vs. 7.26 kg/d, respectively; *P* = 0.05), and 2.5% more when expressed as % of BW (3.24 vs. 3.16% of BW, respectively; *P* = 0.05). Feed efficiency, expressed as gain-to-feed (*P* = 0.69) was not affected by treatment and averaged 0.086 kg.kg^−1^.

**Table 3:** Effects of dietary treatments consisting of two divergent sorghum silages chopped whether at 7.5 mm or 15 mm of theoretical length of cut on the growth performance of angus steers.

	Dietary treatment			
Variable^a^	Sorghum silage at 7.5 mm	Sorghum silage at 15 mm	SEM^b^	*P*-value^c^
**Initial body weight, kg**	205	204	11.1	0.43
**Final body weight, kg**	252	254	2.1	0.46
**Average daily gain, kg**	0.612	0.636	0.0270	0.48
**Dry matter intake, % BW**	3.16	3.24	0.034	0.05
**Dry matter intake, kg/d**	7.26	7.42	0.080	0.05
**Gain to feed, kg/kg**	0.085	0.087	0.0045	0.69

a % BW, expressed as percentage of body weight. Initial and final body weights were collected after 16 hours of feed withdrawal.

b Pooled standard error of treatment, *n* = 6 pens/treatment.

c Observed significance levels for treatment effects.

### Apparent total tract digestibility

**Figure 1:**
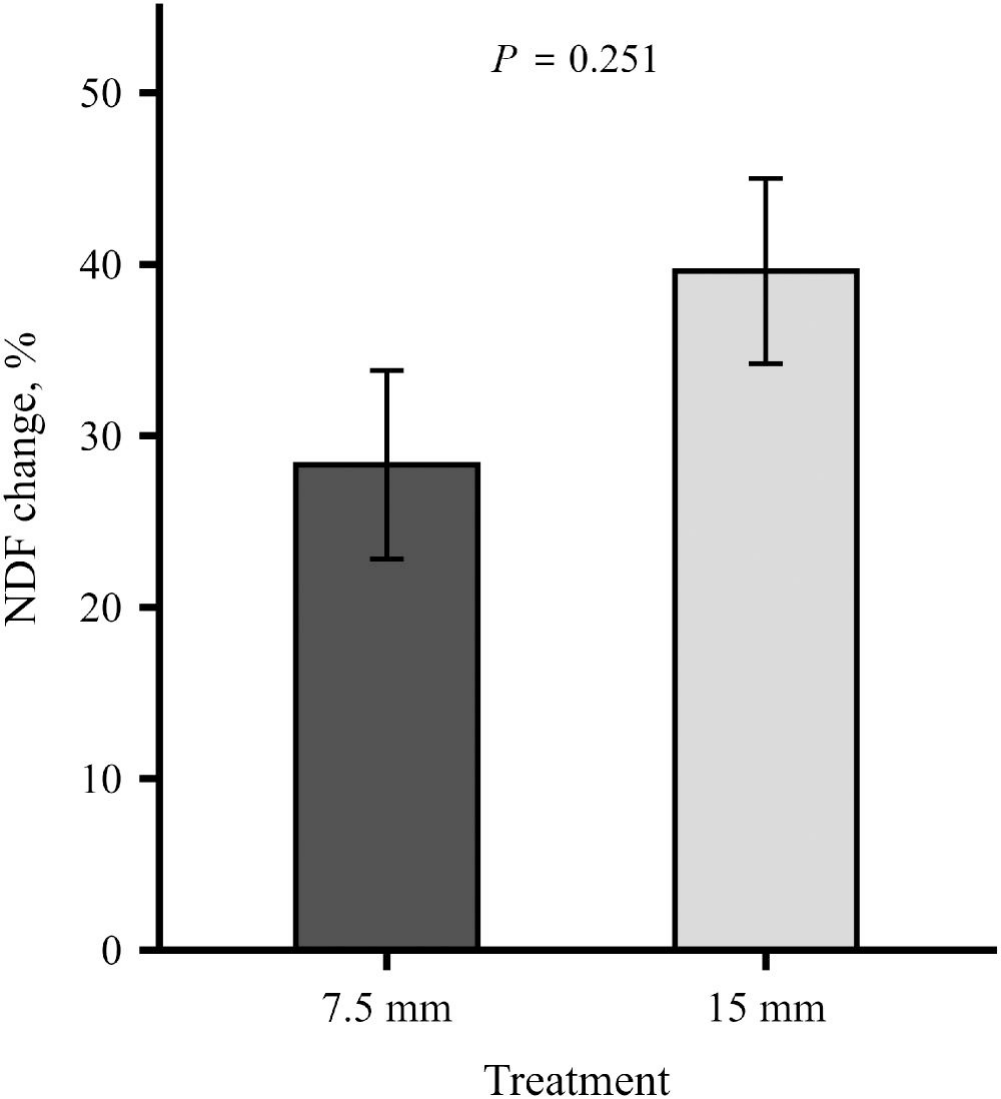
Effects of treatment (7.5 mm vs. 15 mm) on NDF change. Bars represent least squares means; error bars represent ± SEM. Percentage change of NDF concentration = (NDF concentration in orts − NDF concentration diet)/NDF concentration in the diet × 100.

**Figure 2:**
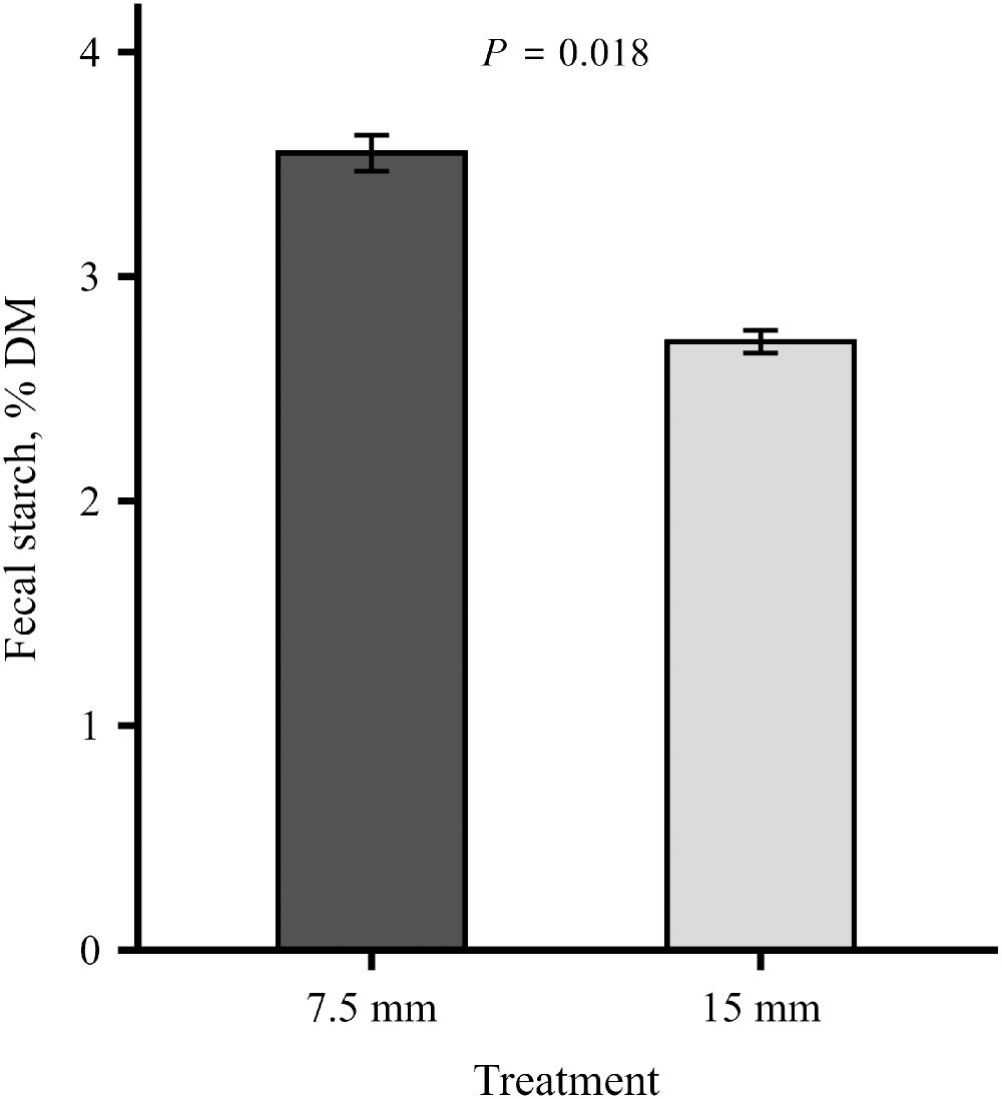
Effects of treatment (7.5 mm vs. 15 mm) on fecal starch. Bars represent least squares means; error bars represent ± SEM.

Digestibility results are shown in Table 4. Dry matter and crude protein intake during the digestibility assessment were not different between treatments (*P* > 0.10). Steers receiving the 15-mm TLOC silage tended to consume more NDF (*P* = 0.06), ADF (*P* = 0.06), and starch (*P* = 0.09) than those receiving the 7.5-mm TLOC silage. No differences were observed in the percentage change of NDF concentration between diet offered and refusals between treatments (Fig. 1; *P* = 0.25). Apparent total tract digestibility of DM, CP, NDF, and ADF did not differ between treatments (*P* > 0.10). However, starch digestibility was greater (*P* = 0.03) in steers fed the 15-mm silage compared with those fed the 7.5-mm silage (92.2% vs. 88.5%, respectively). Fecal starch was lower in steers fed the 15-mm silage vs. those fed the 7.5-mm silage (Fig. 2; *P* = 0.01).

**Table 4:** Apparent total tract digestibility of dietary treatments containing 89% of sorghum silage either chopped at 7.5 mm or 15 mm of theoretical length of cut when fed to angus steers.

	Dietary treatment			
Variable^a^	Sorghum silage at 7.5 mm	Sorghum silage at 15 mm	SEM^b^	*P*-value^c^
***Intake, kg/d***				
**DM**	10.16	9.79	0.470	0.28
**CP**	1.20	1.07	0.086	0.13
**NDF**	5.43	4.94	0.023	0.06
**ADF**	2.87	2.61	0.113	0.06
**Starch**	1.04	1.41	0.105	0.09
***Digestibility, % of intake***				
**DM**	60.7	64.3	1.29	0.13
**CP**	51.2	54.4	2.57	0.52
**NDF**	58.2	58.5	2.6	0.93
**ADF**	54.1	54.0	1.99	0.94
**Starch**	88.5	92.2	0.93	0.03

a DM, dry matter; CP, crude protein; ADF, acid detergent fiber; NDF, neutral detergent fiber.

b Pooled standard error of treatment, *n* = 5 pens/treatment.

c Observed significance levels for treatment effects.

## Discussion

In this experiment, steers fed a diet with sorghum silage chopped at a longer TLOC (15 mm) consumed more dry matter, both in absolute terms and as a percentage of body weight, compared to those fed the diet with sorghum silage chopped at 7.5 mm. This outcome challenges the initial hypothesis that shorter particle sizes would enhance digestibility and thereby stimulate intake and performance. Instead, the increased DMI observed with the coarser silage may reflect improved enhanced physically effective fiber (peNDF), which is known to promote the formation of the rumen mat (Mertens 1997), improving the retention and digestibility of other components. Despite the greater intake, no differences were observed in ADG, final body weight, or feed efficiency between treatments, suggesting that increased consumption did not translate into improved growth performance, likely due to the low energy concentration of the silages which had to be harvested earlier than expected due to sugarcane aphid infestation and an early freeze that precipitated the harvest of the forage with lower starch concentration.

One plausible explanation for the greater starch digestibility observed in the 15 mm TLOC group may involve feed sorting behavior. During the digestibility phase, steers fed the longer-cut silage tended to consume more starch, potentially due to preferential selection of starch-rich particles such as kernels. Miller-Cushon and Devries (2017) and Leonardi and Armentano (2003) demonstrated that cattle sort against long particles and favor finer, fermentable components, which can skew nutrient intake and digestibility. Moreover, Podversich et al. (2025) showed this selective behavior in growing heifers by observing a change in NDF concentration in the diet offered and next-day orts, when growing heifers were fed a 90% sorghum silage diet, either kernel processed or not. However, this behavior is not consistent with the observations in the current study as there were no differences in changes in NDF concentration. Also, the sorting effect was not evident when digestibility was assessed on a dry matter basis as intake of DM was not different between treatments during the digestibility assessment. Although similar patterns have been documented in lactating cows, where increasing forage particle length led to greater selection of fine particles (Leonardi et al. 2005), while reducing particle size typically mitigates sorting behavior, feed intakes during this experiment were maintained at approximately 100% of voluntary intake using a slick-bunk scoring system (Pritchard and Bruns 2003) which would minimize sorting but not rumen fermentation patterns.

Apparent total tract digestibility (ATTD) analyses showed no differences in DM, CP, NDF, or ADF between treatments. However, starch digestibility was notably higher in the 15 mm group (92.2%) compared to the 7.5 mm group (88.5%). This finding was unexpected, given that shorter TLOC are generally associated with improved kernel processing and starch availability (Ferraretto and Shaver 2012; McCary et al. 2020). It is conceivable that the longer chop length facilitated better rumen mat formation and feed stratification, enhancing kernel disruption and microbial fermentation (Allen et al. 2003). Zebeli et al. (2012) proposed that particles larger than 8 mm contribute to the formation of the rumen mat. Sorghum berries are small, dense, and possess a hard, waxy pericarp that resists degradation. For these berries to be digested, they must be retained in the rumen long enough for microbial attachment and fermentation to occur. Functional specific gravity is an important factor influencing particle dynamics in the rumen. Particles with a specific gravity below 1.2 g.ml^−1^ generally remain buoyant and contribute to the rumen mat, whereas those exceeding 1.5 g.ml^−1^ are more likely to sink. As particles are reduced in size, they reach the density and size thresholds for ruminal escape more quickly, which ultimately weakens the structure and consistency of the rumen mat (Allen and Mertens 1988). Particle retention increases with particle size up to 10 mm (Clauss et al. 2011). Beyond this 10 mm threshold, size-dependent retention plateaus as the material becomes extensively entangled within the stratified fiber mat. This entanglement generates a filter-bed effect that facilitates the physical entrapment of denser, less buoyant particles like sorghum berries, thereby delaying their passage from the rumen (Clauss et al. 2011) and potentially improving their digestion. The greater fecal starch concentration observed in the 7.5 mm (Fig. 2) compared with the 15 mm group support this hypothesis.

Despite the greater starch digestibility observed with the longer TLOC, animal performance did not differ between treatments, likely because the overall dietary starch concentration was relatively low. The sorghum silage used in this study contained only 13–14% starch, whereas typical sorghum silages often exceed 20%, suggesting that the magnitude of improvement in starch digestibility may not have been large enough to translate into measurable performance responses.

While literature typically emphasizes the benefits of combining shorter TLOC with aggressive roll-gap settings (eg 1 mm) to maximize starch digestibility, our results suggest that when using conventional sorghum kernel processors, longer TLOC may offer comparable or even superior outcomes in terms of ATTD of starch in growing beef cattle.

Comparable data from Podversich et al. (2025) support this interpretation. Using a conventional processor with a 15 mm TLOC and 1 mm roll gap, those authors reported starch ATTD values near 90%, closely mirroring our findings. In contrast, when TLOC was reduced to 7.5 mm in our study, starch ATTD resembled values typical of unprocessed sorghum silage with a TLOC of 15-mm that those authors observed (88% vs. 86%, respectively). These parallels suggest that the interaction between chop length and processor configuration plays a critical role in starch utilization in beef cattle. In the absence of berry processing score (BPS) data, it is hypothesized that reducing TLOC to 7.5 mm may compromise the efficacy of conventional kernel processors. At very short TLOC like 7.5 mm, the forage mass may behave more fluidly or lack the “traction” necessary for the rollers to exert sufficient shear force on the small sorghum berries, allowing them to pass through the roll gap intact. The 15 mm length may provide the necessary bulk and resistance within the processor to ensure adequate kernel disruption when set at 1 mm roll gap. Future research incorporating BPS is required to confirm whether this mechanical “bypass” explains the digestibility advantage observed at longer chop lengths.

Overall, these results underscore the complex interplay between particle size, and nutrient digestibility in growing beef cattle fed sorghum silage-based diets. While shorter TLOC may enhance fermentation kinetics, our data indicates that a moderate chop length (15 mm) can improve in vivo starch digestibility and support greater feed intake, potentially through enhanced peNDF, mat formation and rumen retention time. In systems lacking aggressive kernel processing or specialized equipment, adopting a 15 mm TLOC may offer a practical advantage, particularly in sorghum-based diets where processor design is crop-specific. This approach could optimize rumen function and starch availability without compromising fiber effectiveness, offering a valuable strategy for improving energy utilization in resource-constrained production environments.

## Conclusion

In growing beef steers fed high-forage diets with whole-plant sorghum silage, a longer theoretical length of cut (15 mm) led to greater dry matter intake and greater starch digestibility compared with a shorter cut (7.5 mm), without impacting ADG or feed efficiency. These results suggest that, in sorghum silage processed with a conventional processor, a moderate chop length may provide a balanced compromise, supporting rumen mat formation while enhancing energy capture. Further research combining chop length with optimized kernel processing using other types of processors is recommended to refine management guidelines for maximizing the nutritive value of whole-plant sorghum silage.

## List of abbreviations

ADF: acid detergent fiber
ADG: average daily gain
ATTD: apparent total tract digestibility
BW: body weight
CP: crude protein
DM: dry matter
DMI: dry matter intake
G:F: gain to feed ratio
GMD: geometric mean diameter
iNDF: indigestible neutral detergent fiber
NDF: neutral detergent fiber
OM: organic matter
peNDF: physically effective neutral detergent fiber
TLOC: theoretical length of cut
